# Pale horse, pale rider done taken my lover away^1^

**DOI:** 10.3201/eid1904.AC1904

**Published:** 2013-04

**Authors:** Polyxeni Potter

**Affiliations:** Author affiliation: Centers for Disease Control and Prevention, Atlanta, Georgia, USA

**Keywords:** art science connection, emerging infectious diseases, art and medicine, flu, 1918 influenza pandemic, Spanish flu, Egon Schiele, Pale horse, pale rider done taken my lover away, expressionism, Self-Portrait with Physalis, about the cover

**Figure:**
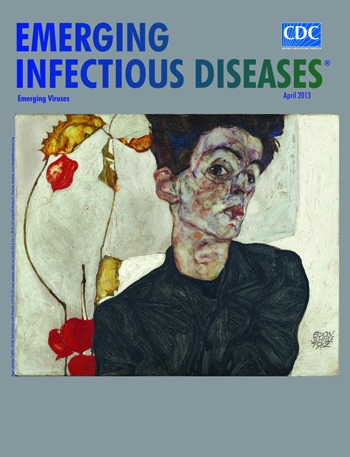
**Egon Schiele (1890–1918) *Self-Portrait with Physalis* (1912) Oil and opaque color on wood (32.2 cm × 39.8 cm)** Leopold Museum, Vienna, Austria, www.leopoldmuseum.org

“It simply divided my life, cut across it like that. So that everything before that was just getting ready, and after that I was in some strange way altered, really,” said Katherine Anne Porter about her nearly fatal encounter with the Spanish flu. “It took me a long time to go out and live in the world again.” Years later, in a thinly disguised autobiographical novel, she laid out not just her own traumatic run-in with death, the pale rider, but also a rare literary account of the 1918 flu pandemic in the United States and the unprecedented human loss.

For her recollection of the pandemic, Porter had to rely on fragments of memory before her illness and after her recovery. These fragments involved the landlady; her beloved fiancé Adam; and fatal flu in Denver, Colorado, during World War I, which killed many young men needed for battle. “I tell you, they must come for her *now* or I’ll put her on the sidewalk.” “They can’t get an ambulance, and there aren’t any beds. And we can’t find a doctor or nurse. They’re all busy.” “It’s as bad as anything can be … all the theaters and nearly all the shops and restaurants are closed, and the streets have been full of funerals all day and ambulances all night.” “Two cherry flavored pills,” “orange juice and ice cream,” “coffee in a thermos bottle.” “The men are dying like flies out there …. This funny new disease. Simply knocks you into a cocked hat.”

Porter wakes up from her illness to find that Adam, “tall and heavily muscled in the shoulders, narrow in the waist and flanks,” handsome Adam with “his eyes pale tan with orange flecks in them, and his hair the color of a haystack when you turn the weathered top back to the clear straw beneath,” Adam, who had “never had a pain in his life that he [could] remember,” had died.

Porter, the consummate storyteller, used words to express the devastating effects of illness on her life. Across the world, in Austria, Egon Schiele, whose self-portrait graces this month’s cover, in similar circumstances, found no comfort in words, despite his own poetic nature. When his wife was dying of the flu, he was unable to articulate his feelings. In a letter to his mother, he coolly speculated that Edith would probably not survive. But he used art to express his devastation. He made several sketches of his wife during the last 2 days of her life. In these sketches, the lines were fluid and sensitive, the colors subdued, the format understated. All these features, a departure from his usual provocative style, reflected the emotional stability found in his life with Edith. His last work was a portrait of his wife, who died the following day. She was 6 months pregnant. He died 3 days later.

Even before his fatal encounter with the flu, Schiele was acquainted with adversity. His early years were marred by a troubled relationship with his mother, poor academic performance, and the loss of his father, a provincial railroad stationmaster, to tertiary syphilis, when Egon was 14 years old. “I don’t know whether there is anyone else at all who remembers my noble father with such sadness.” The elder Schiele had kept his condition from his 17-year-old bride, Egon’s mother. Her first three babies were stillborn. The fourth child died at age 10 of meningitis, a complication of late-onset congenital syphilis. Egon was the first boy to survive. “I shall be the fruit which after its decay will leave behind eternal life; therefore how great must be your joy―to have borne me?” Egon wrote to his mother. His penchant for grandiosity and certain physical features in his early self-portraits led some to wonder whether he might have also been infected.

Schiele lived his life at an accelerated pace. He started to draw as a child and was enrolled in the Vienna Academy of Fine Arts at age 16. He became a protégé of Gustav Klimt, a strong early influence. Once when asked if young Schiele’s drawings showed talent, Klimt responded, “Much too much.” The gifted but troublesome student would soon go off on his own and form the New Artists group. Later, when Klimt was struck down by the flu, Schiele made several portraits of him on his deathbed.

Schiele went on to serve in the military; to have high-profile love affairs; get arrested and be thrown in jail; and before his own untimely death, make a proper and by all accounts promising marriage. All along, he grew as an artist and achieved an expressionist style focused on feelings and their interpretation. He was drawn to the unconventional and controversial, and his hundreds of self-portraits were penetrating and disquieting. His exaggerated lines, unrealistic shapes, and intense colors invoked human situations with a candor that many found disturbing. During his brief career he created more than 3000 works on paper, some 300 paintings. Despite the edginess, his artistic reputation grew, he was offered commissions, and his work sold well.

In interpreting any self-portrait, it is tempting to draw clues from the artist’s life. During his short career, Schiele was alienated and vulnerable and in the midst of World War I and pandemic flu. *Self-Portrait with Physalis*, probably his best-known self-portrait, shows him at the peak of his creativity in 1912, his most productive year, when his expressionist style had matured. Clues for interpreting a self-portrait can also be drawn from facial expressions, gestures, and props within the painting—what the artist allows us to see. In this self-portrait, the arms and body are severely cropped, only the cocked head and shoulders are shown. The one-eyed stare challenges the viewer. So do the pursed lips. This is a laconic composition, though a theatrical one, what with its intensely colored lampion fruit and dreamy sentimentality. Despite the scattered character clues, there is one thing we will never know from Schiele’s *Self-Portrait with Physalis*, and that is what would have happened if he had not died at age 28 of pandemic flu.

When the pandemic ended, which took away even her “decrepit hound and silver kitten,” there was, Porter wrote, a “dazed silence.” The “Great Pandemic” claimed more lives in a short time than any other disease in history, yet because it was intertwined with the “Great War,” the horror of it in human terms may not have been adequately chronicled. Some have called it the “forgotten pandemic”—but not those who work in public health.

Many strides have been made in flu prevention and control since 1918: better understanding of the virus, its distribution in nature, its presence in animals and birds, some of its virulence factors, how it mutates, how it is distributed in tissues, how it is transmitted. We now have prevention programs, vaccines, and antiviral drugs. But many still die, and the threat of another pandemic lurks.

Dramatic tension captured in literature and art prevents us from forgetting the dead and the grave pandemics of history. Schiele did his part by immortalizing the faces of his beloved persons—very much like Porter, who in her version of the spiritual “Pale Horse, Pale Rider,” she contends that death takes away the singer’s lover, mother, siblings, and eventually over the course of several verses the entire family, “But not the singer, not yet,” “Death always leaves one singer to mourn.” That’s to ensure remembrance, which applies as well in public health, where to prevent the next pandemic, it pays to remember and study the past ones.
